# Effect of a Continuous Bedside Pressure Mapping System for Reducing Interface Pressures: A Randomized Clinical Trial

**DOI:** 10.1001/jamanetworkopen.2023.16480

**Published:** 2023-06-02

**Authors:** Chester Ho, Wrechelle Ocampo, Danielle A. Southern, Darlene Sola, Barry Baylis, John M. Conly, David B. Hogan, Jaime Kaufman, Henry Thomas Stelfox, William A. Ghali

**Affiliations:** 1 Division of Physical Medicine & Rehabilitation, Department of Medicine, Faculty of Medicine & Dentistry, University of Alberta and Alberta Health Services Neurosciences, Rehabilitation & Vision Strategic Clinical Network, Edmonton, Alberta, Canada; 2 Department of Medicine, Cumming School of Medicine, University of Calgary, and Alberta Health Services, Calgary, Alberta, Canada; 3 W21C Research and Innovation Centre, O’Brien Institute for Public Health, University of Calgary, Calgary, Alberta, Canada; 4 O’Brien Institute for Public Health, University of Calgary, Calgary, Alberta, Canada; 5 Synder Institute for Chronic Diseases, Cumming School of Medicine, University of Calgary and Alberta Health Services, Calgary, Alberta, Canada; 6 Departments of Critical Care Medicine and Community Health Sciences, University of Calgary, Calgary, Alberta, Canada; 7 Department of Critical Care Medicine, Alberta Health Services, Calgary, Alberta, Canada

## Abstract

**Importance:**

Continuous bedside pressure mapping (CBPM) technology can assist in detecting skin areas with excessive interface pressure and inform efficient patient repositioning to prevent the development of pressure injuries (PI).

**Objective:**

To evaluate the efficacy of CBPM technology in reducing interface pressure and the incidence of PIs.

**Design, Setting, and Participants:**

This parallel, 2-group randomized clinical trial was performed at a tertiary acute care center. The study started to enroll participants in December 2014 and was completed in May 2018. Participants included adults partially or completely dependent for bed mobility. Statistical analysis was performed from September 2018 to December 2022.

**Intervention:**

Nursing staff using visual feedback from CBPM technology for 72 hours.

**Main Outcomes and Measures:**

Absolute number of sensing points with pressure readings greater than 40 mm Hg, mean interface pressure across all sensing points under a patient’s body, proportion of participants who had pressure readings greater than 40 mm Hg, and pressure-related skin and soft tissue changes.

**Results:**

There were 678 patients recruited. After attrition, 260 allocated to the control group (151 [58.1%] male; mean [SD] age, 61.9 [18.5] years) and 247 in the intervention group (147 [59.5%] male; mean [SD] age, 63.6 [18.1] years) were included in analyses. The absolute number of sensing points with pressures greater than 40 mm Hg were 11 033 in the control group vs 9314 in the intervention group (*P* = .16). The mean (SD) interface pressure was 6.80 (1.63) mm Hg in the control group vs 6.62 (1.51) mm Hg in the intervention group (*P* = .18). The proportion of participants who had pressure readings greater than 40 mm Hg was 99.6% in both the control and intervention groups.

**Conclusions and Relevance:**

In this randomized clinical trial to evaluate the efficacy of CBPM technology in the reduction of interface pressure and the incidence of PIs in a tertiary acute care center, no statistically significant benefit was seen for any of the primary outcomes. These results suggest that longer duration of monitoring and adequately powered studies where CBPM feedback is integrated into a multifaceted intervention to prevent PI are needed.

**Trial Registration:**

ClinicalTrials.gov Identifier: NCT02325388

## Key Points

**Question:**

How effective is continuous bedside pressure mapping technology in reducing interface pressure in the inpatient acute care setting?

**Findings:**

In this randomized clinical trial of 678 patients, there was no statistically significant reduction in interface pressure among participants in the intervention group compared with the control group.

**Meaning:**

The results of this trial do not support the use of continuous bedside pressure mapping technology to reduce interface pressures.

## Teaser

This randomized clinical trial investigates the efficacy of continuous bedside pressure mapping technology in reducing interface pressure among patients hospitalized at a tertiary acute care center.

## Introduction

Prevention of pressure injuries (PIs) is a priority for health care systems worldwide. PI incidence and prevalence are widely accepted indicators of quality care. Prevention enhances patient safety^1^ and minimizes expenditures, as preventing PIs is less expensive than treating them.^2^

Frequent repositioning and use of support surfaces are generally accepted strategies for preventing PIs.^3,4^ These measures redistribute interface pressure, which is a major contributing factor to PI development. Although widely used, there is a lack of high-level evidence showing that repositioning reduces the incidence of PIs while pressure-redistributing surfaces have been shown to prevent PIs compared with standard hospital mattresses.^5^ Adequately powered, high-quality trials are needed to determine the optimal frequency and approach to repositioning.^6^

Despite the use of these and other strategies, the overall burden of PIs in hospitalized patients is increasing, presumably related to population aging and increasing prevalence of multiple comorbidities.^7^ Current estimates for the prevalence of PIs globally are 6% to 18.5% in hospital settings, with some countries reporting rates as high as 54%.^8^ PIs annually cost millions of dollars in smaller countries like Portugal (population of approximately 10 million)^9^ and billions in larger countries such as the United States.^10^

Continuous bedside pressure mapping (CBPM) is a technology that monitors interface pressure and bed mobility of patients in real time.^11^ Evidence suggests that CBPM can reduce the risk of PI development.^12^ The technology provides visual feedback that identifies areas of the body with high and/or prolonged pressure exposure. For PI prevention, this technology could either help health care clinicians reposition patients more effectively^11^ or increase the use of surfaces that relieve pressure.^13^

A systematic review by Walia et al^12^ on the efficacy of monitoring devices measuring interface pressure, subdermal tissue stress, motion, and/or moisture in reducing the risk of PIs reported an 88% overall reduction in the risk of developing PIs in 5 of 9 studies identified as using CBPM technology. The authors concluded there was a need for well-controlled, adequately powered prospective cohort studies or randomized clinical trials to determine which technologies are the most effective in reducing the risk of PIs.

Given the dearth of studies evaluating CBPM technology, we conducted a randomized clinical trial that assessed the efficacy of CBPM technology in reducing interface pressure and incident PIs in an acute care setting.

## Methods

### Trial Design

Our hypothesis was that the use of CBPM technology by health care clinicians of patients at high risk of high interface pressures and PI development would decrease these risks. A parallel, 2-group randomized clinical trial (RCT) was conducted at a tertiary acute care center of approximately 1100 beds. Patients at high risk of PI development were recruited from acute general medical, neurology/stroke, neurosurgery, nephrology, and intensive care units (ICU). The primary outcome measures were interface pressures. Secondary measures reported here were pressure-related skin and soft tissue changes (eg, pressure injuries). Participants were randomly assigned to either the intervention or control groups in a ratio of 1:1 stratified by unit. The Research Electronic Data Capture (REDCap) secure database web application hosted at the University of Calgary was used for data storage.^14,15^ Participants were then to be monitored for at least 72 hours. The duration of monitoring was the estimated minimum length of time required to capture the development of PIs in study participants.

Both groups had the CPBM systems placed on their hospital bed to capture interface pressure data. Standard care for PI prevention was provided to both groups. In the intervention group, the monitor was turned on so health care clinicians caring for the participant could visualize the pressure data and target areas of the body requiring pressure relief. The CPBM systems were monitored daily to ensure that they were working. A brief 5-minute educational session was provided to nursing staff 1 to 2 months prior to the initiation of recruitment on the features of the CPBM system and how the data presented on the monitor could be used to reposition patients. Complete information on study design and methods is provided in Wong et al,^16^ although a number of minor modifications to the protocol (Supplement 1) as presented were made that are described here.

This study was approved by the Conjoint Health Research Ethics Board at the University of Calgary prior to data collection. Written informed consent was obtained from all study participants (or legal proxies). This study followed the Consolidated Standards of Reporting Trials (CONSORT) reporting guideline for randomized clinical trials.^17^

### Intervention

The CPBM system used was the ForeSite Patient Turn System (ForeSite PT System), developed by XSENSOR Technologies Inc.^18^ The device is composed of a pressure-sensing mattress cover with 6136 sensing points (ie, sensels) over 52 rows by 118 columns and a monitor that provides visual feedback to health care clinicians. The system captures interface pressure (0-200 mm Hg) at a rate of 1 frame per second. Interface pressure data are transmitted to the monitor and displayed as a colored pressure gradient map of the body. Areas of low pressure are colored blue (eg, <40 mm Hg) with areas of higher pressures colored (in ascending order) yellow, orange, and red. The monitor has a timer to alert health care clinicians when a patient is due for repositioning. The default setting for the timer is 2 hours, but nursing staff are able to adjust this if the patient needs more or less frequent repositioning. A histogram of the patient’s previous 5 turns is on the monitor. This provides information on the time of the previous repositioning and how long it was past due. The identification of body areas on the monitor experiencing high and/or long duration (eg, >2 hours) pressure could then be used by health care clinicians in targeting repositioning efforts. More details are available in a prior publication.^16^

The interface pressure data stored in the monitor were downloaded daily and generated data files in a compressed proprietary binary format. The files were backed up initially on 1 secured server for the first 100 participants, then 2 separate secured servers thereafter. The XSENSOR Pressure Exposure Analyzer Tool was used to export CSV (comma-separated value) formats of the files into an Excel spreadsheet with interface pressure data reduced to 1 frame per minute, which was modified from the original protocol of hourly intervals. The reduction of the data to a frame per minute from a frame per second was supported by correlation coefficient analysis. This modification maintained the fidelity of the data while making it more manageable to analyze.

### Eligibility Criteria

Inclusion criteria were: adults aged 18 years and over; expected length of stay of at least 72 hours; limited bed mobility defined as being either completely (unable to bridge, roll onto their side, and sit from lying supine) or partially (able to bridge or roll onto side, required assistance and/or supervision with sitting from lying supine) dependent based on the de Morton Mobility Index^19^; and, ability to provide consent or the presence of a legal surrogate able to consent. Exclusion criteria were: planned transfer to another unit within 72 hours of enrollment; patients who slept in a chair at night; medical status would have been negatively affected if turned or repositioned; and patients receiving either palliative or end-of-life care with death imminent.

### Recruitment

The research nurse/assistant screened patients newly admitted for eligibility with the nursing staff regularly. Once a patient was identified as potentially eligible, study information was provided to the patient (or legal proxy decision-maker if the patient was not capable of providing consent). Written informed consent was obtained for those who agreed to participate in the study. The participant was then randomized to either the intervention (monitor on) or the control (monitor off) group. The research nurse/assistant set up the CPBM system on the participant’s hospital bed. Participants randomized to the control group had the monitor covered and the settings changed to a low sensitivity for peak pressure (ie, the pressure image even if seen would provide no useful visual feedback). Participants randomized to the intervention group had the monitor uncovered, with the yellow indicator set at 40 mm Hg, orange at 50 mm Hg, and red at 60 mm Hg.

Baseline information collected upon enrollment in both groups were: Braden Score,^20^ skin assessment, participant demographics, and clinical data (ie, Fitzpatrick skin type,^21^ Charlson Comorbidity Index,^22^ de Morton Mobility Index,^19^ body mass index [BMI], bloodwork, admission diagnosis, medical history). Braden scores^20^ for PI risk assessment and clinical skin assessments determined whether there was any preexisting PI.

### Outcomes

The primary outcomes for efficacy assessment were differences between the 2 groups in interface pressures. The pressure measures of interest were: (1) the absolute count of sensels with pressure readings greater than 40 mm Hg; (2) the mean interface pressure across all sensels under the participant’s body (excluding sensels with 0 mm Hg reading); and (3) the proportion of participants who had pressure readings greater than 40 mm Hg. An average pressure of 32 mm Hg was previously referenced as a pressure threshold for tissue damage, but this has not been well validated.^23^ A pressure threshold of 40 mm Hg was selected for this study based on analysis of pilot data that showed a pressure reading of 39.88 mm Hg at the 90th percentile of the distribution of the pressure measured by the ForeSite PT system.^24^

Secondary outcomes consisted of pressure-related skin and soft tissue changes in areas at risk of PI determined on a skin assessment performed by the research nurse after 72 hours and PI risk assessment by Braden score done at baseline and after 72 hours of monitoring. PIs were graded according to the National Pressure Injury Advisory Panel Pressure Injury Stages.^25^

### Statistical Analysis

As per protocol,^16^ 678 participants were enrolled in the trial in order to randomize 339 participants to each of the intervention and control groups. Sample size estimates for the primary outcome indicated a need for 308 participants in each of the groups with an additional 31 participants per group to allow for 10% attrition. This was based on an analysis of pilot data that showed a mean (SD) pressure of 136 (62) mm Hg and allows for the detection of a statistically significant 15% relative decrease in the count of sensels over 40 mm Hg (β, 0.2 [type II error rate]). Univariate statistics were used to summarize changes in pressure distribution as per the interface pressure feedback collected by the CBPM system. Where appropriate, *t* test, analysis of variance, χ^2^, and/or Fisher exact tests were used for comparisons of the primary and secondary outcomes between the control and intervention groups. Standardized mean differences (SMD) were calculated to assess the magnitude and potential importance of any baseline differences in characteristics of the control and intervention groups. A sensitivity analysis was performed for the primary outcome using multiple linear regression, in which adjustment for selected variables, guided by the SMD was performed. SMD greater than 0.10 was used as a threshold to determine which variables would be included in this sensitivity analysis.

Lastly, a non-prespecified subgroup analysis was performed, assessing pressure differences in the intervention vs control group, stratified by ICU vs non-ICU care setting. This analysis was conducted because clinical process observation in the 2 care settings revealed substantial differences in nursing staffing between the 2 settings. It was felt that this difference between settings could lead to differential benefits of the intervention between settings. Two-sided *P* < .05 was considered statistically significant. All statistical analyses were conducted using SAS 9.3 (SAS Institute) from September 2018 to December 2022.

**Figure 1.**
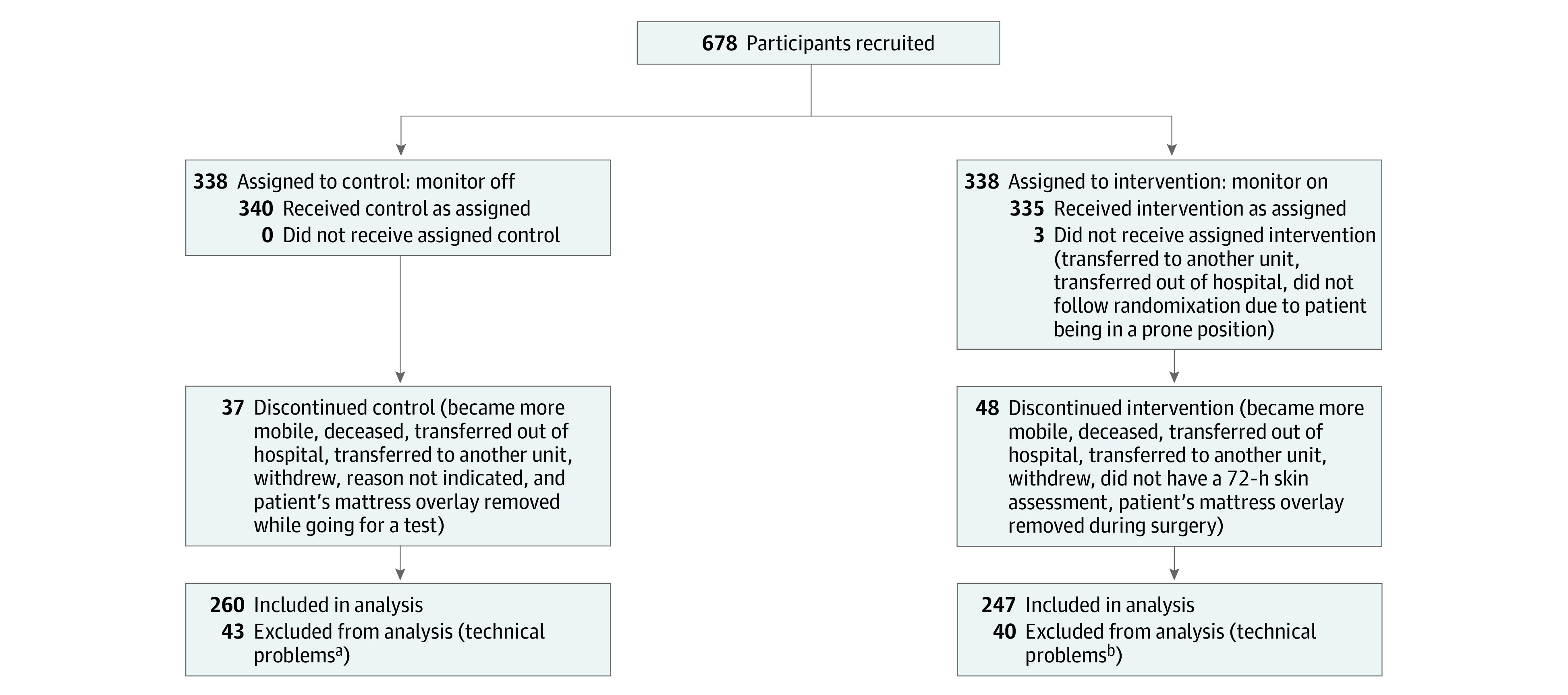
Study Flow Diagram ^a^Technical problems included data that were corrupted on the hard drive and not recoverable (3), data that were not backed up onto a server before the hard drive crashed and were not recoverable (33), files that were not able to be converted to readable data (3), a file that was deleted before being backed up onto a server (1), and less than 72 hours of data (3). ^b^Technical problems included data that were corrupted on the hard drive and not recoverable (10), data that were not backed up onto a server before the hard drive crashed and were not recoverable (29), and a file that was not able to be converted to readable data (1).

Missing clinical data were dealt with by analyzing data using an as-treated approach. Missing pressure readings while the participant was not on the bed for short periods of time (eg, a few hours) during their monitoring were not incorporated into the analysis of the pressure readings. Participants who did not complete approximately 72 hours of monitoring or had technical problems with their pressure data files, as indicated in Figure 1, were excluded from the analysis.

## Results

A total of 678 participants were recruited, of which 340 were randomized to the control group and 338 to the intervention. Eighty of the control participants (23.5%) and 91 of the intervention participants (26.9%) were excluded from analysis. Reasons for exclusion were listed in Figure 1. Among the 260 participants included in the control group, 151 (58.1%) were male; and the mean (SD) age was 61.9 (18.5) years. Among 247 participants included in the intervention group, 147 (59.5%) were male; and the mean (SD) age was 63.6 (18.1) years. Comparisons of baseline characteristics between included and excluded participants showed no significant differences, except for more included participants had diabetes (153 participants [30.3%] vs 38 participants [22.4%]) (eTable 2 in Supplement 2).

The control and intervention groups were well balanced with respect to most baseline characteristics presented in Table 1 (also eTable 1 in Supplement 2), although there were larger differences (ie, larger SMDs) present for 3 variables: paraplegia, solid tumor, and connective tissue disorder. Importantly, the proportion of participants entering the study with a preexisting PI (43% of control participants, 39% of intervention participants) was similar between groups, and there were no significant differences on all categories of the Braden scale.

**Table 1.** Baseline Characteristics of Control vs Intervention Groups

Baseline characteristics	Control, monitor off (n = 260)	Intervention, monitor on (n = 247)	Standardized mean difference
Age, mean (SD), y	61.9 (18.5)	63.6 (18.1)	0.044
Sex, No. (%)			
Female	109 (41.9)	100 (40.5)	0.029
Male	151 (58.1)	147 (59.5)	
Bed mobility, No. (%)			
Dependent	170 (65.1)	153 (61.9)	0.072
Nondependent	89 (34.1)	93 (37.7)	
Missing/not entered	1 (0.4)	1 (0.4)	
BMI, mean (SD)	30.4 (11.0)	32.6 (19.0)	0.099
Weight, mean (SD), kg	86.0 (24.6)	87.9 (29.1)	0.052
Charlson comorbidities, No. (%)			
AIDS/HIV	1 (0.4)	1 (0.4)	0.003
Cerebrovascular disease	115 (44.1)	107 (43.3)	−0.018
Chronic obstructive pulmonary disease	52 (19.9)	58 (23.5)	0.085
Congestive heart failure	65 (24.9)	56 (22.7)	−0.055
Myocardial infarction	33 (12.6)	38 (15.4)	0.078
Dementia	22 (8.4)	24 (9.7)	0.044
Peripheral vascular disease	38 (14.6)	37 (15.0)	0.010
Paraplegia	89 (34.1)	64 (25.9)	−0.182
Leukemia	3 (1.2)	4 (1.6)	0.040
Lymphoma	4 (1.5)	3 (1.2)	−0.028
Peptic ulcer disease	41 (15.7)	42 (17.0)	0.033
Kidney disease	54 (20.7)	48 (19.4)	−0.033
Liver disease	22 (8.4)	17 (6.9)	−0.059
Diabetes	82 (31.4)	71 (28.7)	−0.061
Solid tumor	55 (21.1)	40 (16.2)	−0.128
Connective tissue disease	36 (13.8)	47 (19.0)	0.140
Laboratory values			
Hemoglobin, mean (SD), g/dL	1.02 (.23)	1.02 (0.21)	−0.008
HCT, mean (SD), %	70.0 (8.5)	60.0 (2.8)	−0.014
RBC, mean (SD), 10 × 10^6^/dL	3.8 (5.2)	3.4 (0.8)	−0.070
Platelet count, mean (SD), 10 × 10^3^/dL	248.5 (145.0)	239.6 (130.9)	−0.046
Creatinine, mean (SD), mg/dL	1.24 (1.29)	1.35 (1.35)	0.056
WBC, mean (SD), 10 × 10^3^/dL	11.8 (8.7)	12.2 (16.8)	0.020
Braden score, mean (SD)	13.47 (2.19)	13.50 (1.95)	0.010
Pressure injury status			
No PI at baseline, No. (%)	149 (57.3)	152 (61.5)	0.086
PI positive at baseline, No. (%)	111 (42.7)	95 (38.5)	
No. of areas at baseline, No.	111	95	
1 area, No. (%)	62 (55.9)	51 (53.7)	0.078
2 areas, No. (%)	24 (21.6)	21 (22.1)	
3 areas, No. (%)	14 (12.6)	17 (17.9)	
≥4 areas, No. (%)	11 (9.9)	6 (6.2)	

Abbreviations: HCT, hematocrit; RBC, red blood cell; WBC, white blood cell.

SI unit conversion factors: To convert creatinine to μmol/L, multiply by 88.4; to convert hemoglobin to g/L, multiply by 100.0; to convert platelet count to ×10^3^/μL, multiply by 1.0; to convert RBC to ×10^12^/L, multiply by 1.0; to convert WBC to ×10^9^/L, multiply by 1.0.

Table 2 presented data on the primary and secondary outcomes. Overall, the absolute number of sensels with pressures greater than 40 mm Hg was 11 033 in the control group vs 9314 in the intervention group (*P* = .16). The mean (SD) pressure in the control group was 6.80 (1.63) mm Hg vs 6.62 (1.51) mm Hg in the intervention group (*P* = .18).

**Table 2.** Primary and Secondary Outcomes^a^

Outcomes	Overall		
	Control, monitor off (n = 260)	Intervention, monitor on (n = 247)	*P* value
Primary outcomes			
Peak pressure 72 h	256.0	256.0	NA
Absolute number of sensels with pressure readings >40 mm Hg, No.	11 033	9314	.16^b^
Pressure in mm Hg, mean (SD)	6.80 (1.63)	6.62 (1.51)	.18^c^
Proportion of participants who have pressure readings >40 mm Hg, No. (%)	259 (99.6)	246 (99.6)	.97^c^
Count of sensels with pressure >40 mm Hg, median (IQR)	27.9 (18.1-37.7)	26.2 (17.9-34.5)	.19^d^
Pressure in mm Hg, median (IQR)	6.56 (6.11-7.01)	6.26 (5.82-6.70)	.10^d^
Secondary outcomes			
No pressure injury at baseline, No.	149	152	NA
Pressure injury at 72 h, No. (%)	23 (15.4)	31 (20.4)	.26^b^
Pressure injury at baseline & at 72 h, No.	111	95	NA
New location at 72 h, No. (%)	29 (26.1)	10 (10.5)	.004^b^

Abbreviation: NA, not applicable.

^a^ Only nonzero values included in calculations.

^b^ Calculated using χ^2^ test.

^c^ Calculated using *F*-test.

^d^ Calculated using Kruskal-Wallis test.

As aforementioned, there were somewhat larger baseline differences between the intervention and control groups for 3 variables: paraplegia, solid tumor, and connective tissue disorder (each with SMD >0.10). A multivariable linear regression controlling for these 3 variables was essentially unchanged from the unadjusted analysis, with no appreciable effect on either the statistical significance of between-group differences or the β coefficient of the pressure difference between the intervention and control groups.

In the non-prespecified subgroup analysis comparing outcomes in the intervention and control groups, stratified by ICU vs non-ICU care setting, we observed a median (IQR) pressure difference of 0.39 (0.37-0.41) for non-ICU care vs 0.15 (0.12-0.18) in the ICU care setting (*P* = .22).

**Figure 2.**
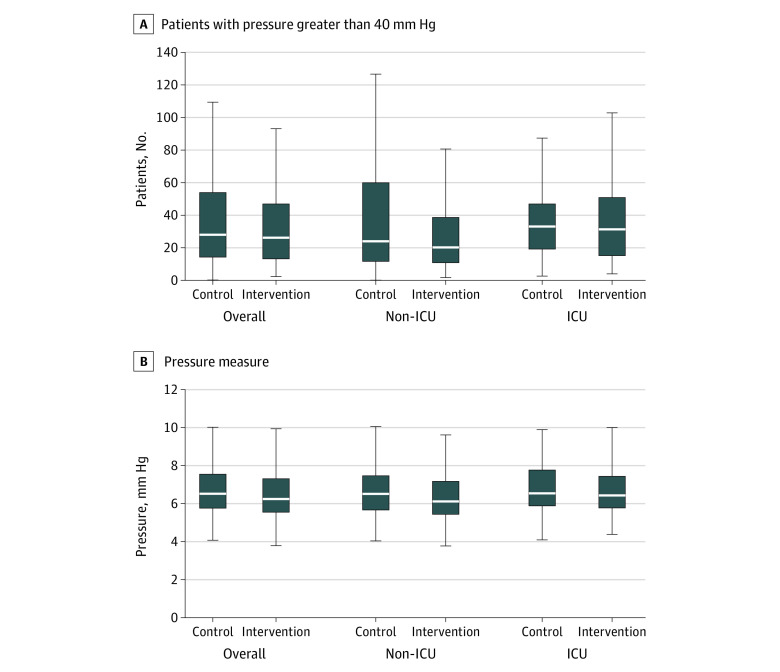
Box Plots for Median Count of Sensels Greater Than 40 mm Hg and Median Pressure Averaged Over All Time Overall results are presented in addition to non-prespecified subgroup analysis of ICU vs non-ICU location of care. The horizontal lines dividing the groups are called quartiles, and the groups are referred to as quartile groups. The middle line is the median, top and bottom are upper and lower quartiles, respectively. The middle box represents the middle 50% of scores for the group. The range of scores from lower to upper quartile is the IQR. The upper and lower whiskers represent scores outside the middle 50%. The lowest is the lowest data point in the data set excluding any outliers, and the highest is the highest data point in the data set excluding any outliers. ICU indicates intensive care unit.

Box plots for both primary outcomes and subgroup analyses are presented in Figure 2. Medians were presented due to skewness of the data. Overall, there was no difference in the presence of PIs at baseline or 72 hours, but there was a statistically significant difference in the appearance of new skin changes at different locations among those who had a PI at baseline (26.1% of control group vs 10.5% of intervention group; *P* = .004). This was driven primarily by the difference found in the non-ICU group (22% of control group vs 5% of intervention group; *P* = .005) (eTable 3 in Supplement 2).

## Discussion

Our RCT evaluating CBPM technology followed earlier published studies on the same technology.^24,26^ We examined how the technology affected interface pressure parameters and, ultimately, the risk of PI development.

Prior CBPM studies typically focused on either clinical^27^ or interface pressure^26^ outcomes rather than both concurrently. Prior ICU studies, in particular, have shown promising results. Behrendt et al^27^ in a RCT showed that use of CBPM was associated with a significant reduction in hospital-acquired PIs in a medical ICU. Grap et al^26^ studied the effects of CBPM on interface pressures at 7 anatomical locations in ICU patients, while Siddiqui et al^28^ compared the rates of PI development between a group of patients using CBPM in a medical ICU with historical controls. Gunningberg et al^29^ reported on both interface pressure and clinical outcomes. In contrast to our study, their setting was a geriatric internal medical hospital ward where red areas on the pressure image indicated pressures of 75 mm Hg or greater. They found no difference in prevalence or incidence of PIs or peak pressures between the groups. Although our study was designed to be adequately powered to detect a 15% relative reduction in the interface pressure primary end point, it ultimately was underpowered due to unforeseen technical issues with data loss and due to the diversity of nursing units involved. This may explain the lack of statistical difference in the primary outcomes between the control and intervention groups.

Notwithstanding the lack of statistical significance on measures of central tendency (Table 2), the box plots presented in Figure 2 indicate a modest reduction in patients with high interface pressures and counts of sensels with pressure over 40 mmHg, especially for those in the non-ICU settings. Although the clinical importance of these interface pressure differences is uncertain, the corresponding difference seen in the secondary outcome of skin changes at 72 hours among patients with a baseline PI (Table 2) is notable.

Taken as a whole, the findings of this clinical trial are mixed. The primary outcomes do not differ significantly between groups. For the primary end point, it is indeed unfortunate that data loss resulted in the study being underpowered to detect a statistically significant 15% relative reduction in the pressure measures (particularly because the relative difference in the study was 16% for the primary outcome). Alongside this, one of the secondary outcomes (skin changes at new locations) was reduced in the intervention group. Lastly, the ICU vs non-ICU subgroup analysis, which importantly was not a prespecified subgroup analysis, raises the possibility (which should only be viewed as a hypothesis-generating possibility) that the CBPM intervention may be most beneficial in care settings where there is less nurse staffing and lower nursing care intensity.

Our study findings must be interpreted in the context of existing literature on CBPM systems. Collectively, studies do suggest that CBPM may be a useful component of multifaceted efforts to decrease new PIs among those at the highest risk. Our findings are consistent with previous results^24^ that interface pressures may be reduced with use of this technology, especially in higher risk settings. It is not clear what underlies this potential beneficial effect of CBPM, although based on our earlier work^24^ we suspect it is due to more frequent and directed repositioning that relieves pressure on at-risk areas. These findings along with previous studies^12,29^ support the need for larger-scale RCTs of longer duration to further examine this technology. In particular, these larger-scale RCTs will be most useful if they can be sufficiently powered for the hard clinical end points that matter most.

We anticipate that CBPM systems will be most effective if incorporated as part of a multicomponent intervention including staff training on PI prevention and the use of pressure-reducing mattresses, which was also considered in the study by Gunningberg et al.^29^ The non–statistically significant differential finding in our study of possibly greater benefit in non-ICU settings relative to ICU (Table 2), where multifaceted PI prevention and mitigation strategies are often already in place,^30,31,32^ are supportive of bundled multicomponent approaches. It may be particularly fruitful to explore the use of CBPM technology in other care settings where there is a concentration of patients at risk of pressure injury development and yet lower nursing staff to patient ratio (ie, settings such as inpatient rehabilitation hospitals, skilled nursing facilities, long-term care facilities and long-term acute care hospitals).

### Limitations

There are several limitations to our study. First, we had greater data attrition than expected due to data loss from storage failure in the downloading of data from the CBPM system at the start of our study. This unanticipated data loss underscores the need for research teams to secure robust data back-up in future studies of this technology, especially when terabytes of data are being produced, as was the case in this study. As a result of this data loss, our study was underpowered (post hoc calculated power 52%), which might explain the non–statistically significant outcomes. We were also underpowered to detect new-onset PIs due to the relatively low incidence of PI at 72 hours. To address this challenge of statistical power to study hard clinical end points will probably require a multicenter study with a much larger sample size and longer follow-up. A second limitation is that improved nursing interventions at the bedside, such as avoidance of 90-degree lateral positioning, could have also contributed to more stable pressure profiles,^33^ the low incidence of PIs, and lack of difference found between the control and intervention groups. A third limitation is that the pressure outcomes examined were summary measures that do not give data on the specific locations where PIs tend to develop, nor provide a portrayal of the full duration of the interface pressure. A fourth limitation pertaining to the secondary outcome assessment is that skin changes indicative of PI can vary in the same person from day to day.^29,33^ This may lead to sampling bias and/or error in assessing clinical PI outcomes over relatively short periods of time, which could have contributed to difficulties in assessing differences between the 2 groups. Additionally, there is the possibility of a Hawthorne effect where conducting the study may have elevated the overall attention paid to PIs and their prevention. Both intervention and control participants were on the same units. Such a Hawthorne effect would possibly bias the study toward a null result, although our randomization process should have mitigated this potential effect.

Weighing against these limitations are some clear strengths. This was a substantial RCT assessing the efficacy of a CBPM system on interface pressures as a risk factor for PI development. We also used clinically relevant outcomes juxtaposed to the pressure sensels readings to add correlative measurements. The study was not funded by the device manufacturer, but rather through competitive peer-reviewed public sector funding. Furthermore, our research team had no affiliation with the CBPM system manufacturer.

## Conclusions

In this randomized clinical trial of the efficacy of CBPM in a single tertiary acute care hospital, we did not show any statistically significant benefit in the reduction of interface pressure. Future research on CBPM studies should include longer duration of monitoring, adequately powered sample size for both pressure and clinical end points, and an economic evaluation of the use of this technology.

## Notes

Supplement 1.
Trial Protocol

Supplement 2. **eTable 1.** Other Baseline Characteristics of Control vs. Intervention Groups **eTable 2.** Baseline Characteristics of Patients Included vs. Patients Included in Interface Pressure Analysis **eTable 3.** Non-Pre-Specified Subgroup Analysis of Primary and Secondary Outcomes Stratified by ICU vs. Non-ICU Location of Care

Supplement 3.
Data Sharing Statement

## References

[1] Sammon M, Dunk A, Verdu J. Advances in pressure ulcer prevention and treatment. Wounds International. Published November 5, 2015. Accessed July 2, 2020. https://www.woundsinternational.com/resources/details/advances-pressure-ulcer-prevention-and-treatment-made-easy

[2] Schuurman JP, Schoonhoven L, Defloor T, van Engelshoven I, van Ramshorst B, Buskens E. Economic evaluation of pressure ulcer care: a cost minimization analysis of preventive strategies. Nurs Econ. 2009;27(6):390-400, 415. 20050490

[3] Haesler H, ed. Prevention and treatment of pressure ulcers/injuries: quick reference guide. European Pressure Ulcer Advisory Panel, National Pressure Injury Advisory Panel and Pan Pacific Pressure Injury Alliance. Updated 2019. Accessed April 28, 2023. https://www.internationalguideline.com/static/pdfs/Quick_Reference_Guide-10Mar2019.pdf

[4] Norton L, Parslow N, Johnston D, . Best practice recommendations for the prevention and management of pressure injuries. foundations of best practice for skin and wound management. Wound Care Canada. Published 2017. Accessed April 28, 2023. http://www.woundscanada.ca/ docman/public/health-care-professional/bpr-workshop/172-bpr-prevention-andmanagement-of-pressure-injuries-2/file

[5] McInnes E, Jammali-Blasi A, Bell-Syer SEM, Dumville JC, Middleton V, Cullum N. Support surfaces for pressure ulcer prevention. Cochrane Database Syst Rev. 2015;2015(9):CD001735. doi:10.1002/14651858.CD001735.pub5 26333288 PMC7075275

[6] Gillespie BM, Chaboyer WP, McInnes E, Kent B, Whitty JA, Thalib L. Repositioning for pressure ulcer prevention in adults. Cochrane Database Syst Rev. 2014;2014(4):CD009958. doi:10.1002/14651858.CD009958.pub2 24700291 PMC6769133

[7] Boyko TV, Longaker MT, Yang GP. Review of the current management of pressure ulcers. Adv Wound Care (New Rochelle). 2018;7(2):57-67. doi:10.1089/wound.2016.0697 29392094 PMC5792240

[8] Tubaishat A, Papanikolaou P, Anthony D, Habiballah L. Pressure ulcers prevalence in the acute care setting: a systematic review, 2000-2015. Clin Nurs Res. 2018;27(6):643-659. doi:10.1177/1054773817705541 28447852

[9] Silva AJ, Pereira SM, Rodrigues A, . Economic cost of treating pressure ulcers: a theoretical approach. Rev da Esc Enferm. 2013;47(4):967-972. doi:10.1590/S0080-623420130000400028 24310698

[10] Markova A, Mostow EN. US skin disease assessment: ulcer and wound care. Dermatol Clin. 2012;30(1):107-111, ix. doi:10.1016/j.det.2011.08.005 22117872

[11] Scott RG, Thurman KM. Visual feedback of continuous bedside pressure mapping to optimize effective patient repositioning. Adv Wound Care (New Rochelle). 2014;3(5):376-382. doi:10.1089/wound.2013.0519 24804157 PMC4005484

[12] Walia GS, Wong AL, Lo AY, . Efficacy of monitoring devices in support of prevention of pressure injuries. Adv Skin Wound Care. 2016;29(12):567-574. doi:10.1097/01.ASW.0000504579.83707.f6 27846030

[13] Edwards JA, Scheider SL, Thurman KM. Enhancing support surface therapeutics and decreasing user error using continuous bedside pressure mapping. Poster presented at: Symposium on Advanced Wound Care; April 2014; Orlando, FL.

[14] Harris PA, Taylor R, Minor BL, ; REDCap Consortium. The REDCap consortium: building an international community of software platform partners. J Biomed Inform. 2019;95:103208. doi:10.1016/j.jbi.2019.103208 31078660 PMC7254481

[15] Harris PA, Taylor R, Thielke R, Payne J, Gonzalez N, Conde JG. Research electronic data capture (REDCap)–a metadata-driven methodology and workflow process for providing translational research informatics support. J Biomed Inform. 2009;42(2):377-381. doi:10.1016/j.jbi.2008.08.010 18929686 PMC2700030

[16] Wong H, Kaufman J, Baylis B, . Efficacy of a pressure-sensing mattress cover system for reducing interface pressure: study protocol for a randomized controlled trial. Trials. 2015;16:434. doi:10.1186/s13063-015-0949-x 26420303 PMC4588270

[17] Butcher NJ, Monsour A, Mew EJ, . Guidelines for Reporting Outcomes in Trial Reports: The CONSORT-Outcomes 2022 Extension. JAMA. 2022;328(22):2252-2264. doi:10.1001/jama.2022.21022 36511921

[18] XSENSOR Technology Corporation. ForeSite PT System. Accessed April 28, 2023. https://www.xsensor.com/patient-monitoring

[19] de Morton NA, Davidson M, Keating JL. The de Morton Mobility Index (DEMMI): an essential health index for an ageing world. Health Qual Life Outcomes. 2008;6(1):63. doi:10.1186/1477-7525-6-63 18713451 PMC2551589

[20] Bergstrom N, Braden BJ, Laguzza A, Holman V. The Braden Scale for predicting pressure sore risk. Nurs Res. 1987;36(4):205-210. doi:10.1097/00006199-198707000-00002 3299278

[21] Fitzpatrick TB. The validity and practicality of sun-reactive skin types I through VI. Arch Dermatol. 1988;124(6):869-871. doi:10.1001/archderm.1988.01670060015008 3377516

[22] Charlson M, Szatrowski TP, Peterson J, Gold J. Validation of a combined comorbidity index. J Clin Epidemiol. 1994;47(11):1245-1251. doi:10.1016/0895-4356(94)90129-5 7722560

[23] Bader D, Oomens C. Recent Advances in Pressure Ulcer Research. In: Romanelli M, Clark M, Cherry G, Colin D, Defloor T, eds. Science and Practice of Pressure Ulcer Management. Springer; 2006. doi:10.1007/1-84628-134-2_3

[24] Motamedi SM, de Grood J, Harman S, . The effect of continuous pressure monitoring on strategic shifting of medical inpatients at risk for PUs. J Wound Care. 2012;21(11):517-518, 520, 522 passim. doi:10.12968/jowc.2012.21.11.517 23413490

[25] Edsberg LE, Black JM, Goldberg M, McNichol L, Moore L, Sieggreen M. Revised national pressure ulcer advisory panel pressure injury staging system: revised pressure injury staging system. J Wound Ostomy Continence Nurs. 2016;43(6):585-597. doi:10.1097/WON.0000000000000281 27749790 PMC5098472

[26] Grap MJ, Munro CL, Wetzel PA, . Tissue interface pressure and skin integrity in critically ill, mechanically ventilated patients. Intensive Crit Care Nurs. 2017;38:1-9. doi:10.1016/j.iccn.2016.07.004 27836262 PMC5641974

[27] Behrendt R, Ghaznavi AM, Mahan M, Craft S, Siddiqui A. Continuous bedside pressure mapping and rates of hospital-associated pressure ulcers in a medical intensive care unit. Am J Crit Care. 2014;23(2):127-133. doi:10.4037/ajcc2014192 24585161

[28] Siddiqui A, Behrendt R, Lafluer M, Craft S. A continuous bedside pressure mapping system for prevention of pressure ulcer development in the medical ICU: a retrospective analysis. Wounds. 2013;25(12):333-339. doi:10.1016/j.enfi.2010.10.003 25867745

[29] Gunningberg L, Sedin I-M, Andersson S, Pingel R. Pressure mapping to prevent pressure ulcers in a hospital setting: a pragmatic randomised controlled trial. Int J Nurs Stud. 2017;72:53-59. doi:10.1016/j.ijnurstu.2017.04.007 28460263

[30] Tayyib N, Coyer F. Effectiveness of pressure ulcer prevention strategies for adult patients in intensive care units: a systematic review. Worldviews Evid Based Nurs. 2016;13(6):432-444. doi:10.1111/wvn.12177 27712030

[31] Swafford K, Culpepper R, Dunn C. Use of a comprehensive program to reduce the incidence of hospital-acquired pressure ulcers in an intensive care unit. Am J Crit Care. 2016;25(2):152-155. doi:10.4037/ajcc2016963 26932917

[32] Sharma K, Hastings SE, Suter E, Bloom J. Variability of staffing and staff mix across acute care units in Alberta, Canada. Hum Resour Heal 2016 141. 2016;14(1):1-8. doi:10.1186/s12960-016-0172-1 PMC5131495 27903297

[33] Kim SY, Shin YS. A comparative study of 2-hour interface pressure in different angles of laterally inclined, supine, and fowler’s position. Int J Environ Res Public Health. 2021;18(19):9992. doi:10.3390/ijerph18199992 34639293 PMC8507824

