# Supplementary material for: Effect of a Continuous Bedside Pressure Mapping System for Reducing Interface Pressures: A Randomized Clinical Trial
Source: JAMA Netw Open. 2023 Jun 2;6(6):e2316480. doi: 10.1001/jamanetworkopen.2023.16480 (PMC10238950; doi:10.1001/jamanetworkopen.2023.16480)
Supplement: Supplement 1. — Trial Protocol [file jamanetwopen-e2316480-s001.pdf]

Effectiveness of a pressure-sensing mattress system for reducing the risk of pressure ulcer formation by lowering interface pressure in vulnerable patient populations: Parallel-two-group randomized trial

Abstract

Background: Pressure ulcers are a significant challenge in healthcare, resulting in substantial financial costs to hospitals and personal costs borne by affected patients and their families. Continuous pressure imaging (or interface pressure mapping) of the interface pressure between the body and support surface could be an effective technology as part of an overall strategy for the prevention and management of ulcers. This randomized controlled trial builds on previous research to establish the effectiveness of XSENSOR Technology Corporation's ForeSite PT™ system, a continue pressure imaging technology, in its ability to reduce interface pressure and reduce the incidence of pressure ulceration.

Methods: A parallel two group randomized controlled clinical trial will be conducted. A total of 600 eligible consenting inpatients at a tertiary care institution will be randomly allocated to either have the ForeSite PT™ system and the LCD monitor turned on (intervention group), or have the ForeSite PT™ system and the LCD monitor turned off (control group) in a ratio of 1:1. Both groups will be included in the study up to a maximum of 7 days. In addition to interface pressure, clinical outcomes will also be measured at baseline (within 24 hours of admission) and on the last day of enrollment in the trial (within 24 hours prior to discharge, or on the seventh day (whichever comes first)). The perceptions of patients allocated to the intervention group, and the perceptions of healthcare providers each time a patient in their care is allocated to the intervention group, will be measured on the last day of enrollment in the trial or on the seventh day (whichever comes first).

Discussion: This will be the first randomized controlled trial to investigate the effectiveness of continuous pressure imaging on pressure management among, and the association between interface pressure and development of pressure-related skin and soft tissue change with potential for progression to overt pressure ulceration. The results could provide evidence to inform clinical practice guidelines on the prevention and management of pressure ulcers.

Trial registration: TBD

Keywords: Pressure ulcers, Bedsores, Continuous pressure mapping, Interface pressure imaging

Background

Pressure ulcers are a leading cause of morbidity (and in some instances, mortality) in hospitalized and institutionalized patients (Woodbury), resulting in substantial financial costs to hospitals and personal costs borne by affected patients and their families. A pressure ulcer is "a localized injury to the skin and/or underlying tissue over a bony prominence, as a result of pressure, or pressure in combination with shear (NPUAP)." The size and severity of pressure ulcers vary, ranging from skin erythema to full thickness tissue loss with damage extending into muscle and bone (NPUAP Staging). Annually, there are

an estimated 250,000 to 500,000 individuals affected by pressure ulceration in Canada, with the overall estimated prevalence in all health-care institutions to be 26.0% in Canada (Dealey, Wound Care in Canada).

Pressure ulcer development and recurrence is multi-factorial; however, they primarily occur in people who have limited mobility, nerve damage, or both. Risk factors include limited mobility and activity; perfusion (including diabetes); skin conditions; skin moisture and incontinence; advanced age; reduced levels of albumin, haemoglobin/anaemia, lymphocytes, and other blood serum components; compromised nutritional status; and general health status (Coleman, Dorner). Other predictors of pressure ulceration include ICU admission length, norepinephrine administration, and cardiovascular disease among adult critical care patients (Cox), while delayed and inappropriate medical management, history of or existing pressure ulceration, and limited independence in self-care were also predictors among adults with spinal cord injuries (Marin).

The economic impact of pressure ulcers is significant, as the estimated cost of treatment in the UK varies from £1,214 to £14,108 per case. The cost of treatment increases with severity as more severe cases require longer healing times and have higher incidence of complications (Dealey). The financial burden of pressure ulcers are also significant in the United States, where the average hospital treatment cost associated with stage IV pressure ulcers and related complications was $129,248 for one hospital admission, and $124,327 for those acquired in the community over four hospital admissions (Brem). In Canada, the estimated average monthly cost of pressure ulcer management among individuals with spinal cord injury was $4,475, with hospital admission costs making up the greatest proportion of the total cost (Chan). The cost of nursing time, which is comprised of dressing the wound, repositioning the patient and monitoring and risk assessment, amount to the vast majority of resource cost of treatment (Dealey).

Many pressure ulcers are avoidable; however, it is generally accepted that there are situations where they cannot be prevented (Black, Langemo, NPUAP). There are various modalities to prevent and treat pressure ulcers, including wound cleansers, repositioning, negative pressure therapy, debridement, enteral and parenteral feeding, vitamin and mineral supplementation, specialized mattresses, support services, ultrasound therapy, honey, cellular therapy, musculocantaneous and fasciocutaneous flap closure; however, there is no widely accepted formal process for treatment (Levine). Rather, many acute care settings and long-term-care facilities adopt multi-pronged, multidisciplinary interventions to prevent pressure ulcers (Niederhauser). Current guidelines advise health care practitioners to conduct a standardized pressure ulcer risk assessment (Toolkit, SCI Guideline). The use of risk assessment scales provides a more accurate prediction of pressure ulcer risks than clinical judgment (Pancorbo-Hidalgo). The Braden Scale for Predicting Pressure Sore Risk (Bergstrom) is recommended as it has received the most validation and has demonstrated the best sensitivity/specificity balance and the highest predictive capacity (Pancorbo-Hidalgo). As applied pressure over a prolonged period is a primary cause of pressure ulcers, frequent and regular repositioning of the patient has been recommended as an integral

component of prevention and treatment protocols. The best practices pertaining to a specific turning regiment varies from the National Pressure Ulcer Advisory Panel (NPUAP) recommending repositioning every two hours and the European Pressure Ulcer Advisory Panel (EPUAP) recommending every four hours. Thus pressure ulcer prevention and management are necessarily labour-intensive.

Continuous pressure imaging (or interface pressure mapping) of the interface pressure between the body and support surface could be an effective technology as part of an overall strategy for the prevention and management of ulcers. By placing a thin mattress or sensor pad with monitoring capabilities under a patient, pressure imaging interprets the interface pressures and provides the patient's real-time pressure points on a corresponding computer screen, which can assist healthcare providers determine when a patient should be re-positioned (Hanson). XSENSOR Technology Corporation designed the ForeSite PT™ Patient Turn System (here in "ForeSite PT™ system"), which can continuously monitor bed surface interface pressure and track the patient's exposure to that pressure. The ForeSite PT™ system provides patient turn tracking to assist with management of the patient turn schedule. The pressure exposure monitor provides a visual image that quantifies historical pressure information. The exposure monitor assists clinical staff by alerting them to the location of body areas that have experienced the greatest exposure to pressure. The surface pressure monitor provides clinical staff with a visual image of the pressure distribution across a patient's body. It can be used by care providers to identify high pressure areas in real time and to validate that patient repositioning has successfully relieved pressure.

The ForeSite PT™ system emerged, in part, from a partnership between the Ward of the 21st Century (W21C) and XSENSOR to support the development and commercialization of this technology. This project included focus groups; 1-1 meetings with clinicians, W21C leads and managers; usability testing sessions; heuristic evaluations for iterative technology development; a pilot and a natural history study; and concluded with the evaluation of the ForeSite PT™ system through focus groups, usability testing, and a pilot study. The proposed study will build on this previous research conducted with XSENSOR to establish the effectiveness of a pressure-sensing mattress system (ForeSite PT™ system).

The ForeSite PT™ system consists of two parts: a thin, flexible sensor overlay that is positioned under the hospital linen (under the fitted sheet) and an LCD monitor that is mounted to the headwall (and required an electrical outlet). The LCD monitor displays colour information about patient surface pressure on the bed and provides risk information determined by the length of time pressure had persisted in any location.

Objectives

The primary objective of this study is to:

1. establish the effectiveness of a pressure-sensing mattress system (ForeSite PT™ system) in its ability to reduce interface pressure as reflected in a composite of pressure measurements.

The secondary objectives are to:

1. assess whether the ForeSite PT™ system reduces the incidence of pressure-related compromised skin and soft tissues, with potential for progression to overt pressure ulceration;
2. determine the minimum interface pressure that is associated with higher risk for pressure ulcer development through the correlation of clinical outcomes and pressure outcomes;
3. assess healthcare provider and patient perceptions of the ForeSite PT™ system; and
4. establish the cost-effectiveness of the ForeSite PT™ system.

Specifically, the study will address the following research questions:

1. Is the ForeSite PT™ system effective at reducing interface pressure by assisting healthcare providers in determining the best method for patient turning/repositioning?
2. Does the use of the ForeSite PT™ system reduce the incidence of pressure-related compromised skin and soft tissues, with potential for progression to overt pressure ulceration?
3. Does interface pressure correlate with clinical outcomes?
4. Does the ForeSite PT™ system have more impact on the clinical outcomes of patients who have been identified as having a higher risk of pressure ulceration as per the Braden scale?
5. What are the perceptions (both positive and negative) of healthcare providers to the ForeSite PT™ system with regards to functionality, ease of use, and interpretation of pressure data on the computer monitor?
6. What are the perceptions (both positive and negative) of patients (and if appropriate, family members) to the ForeSite PT™ system (including sensor pad and monitor display of their pressure points)?

Methods

Trial design

A parallel two group randomized controlled clinical trial will be conducted to assess the use of ForeSite PT™ system on a pressure-related skin and tissue changes, with progression to pressure ulceration; interface pressure; and the perceptions of healthcare providers and patients to the ForeSite PT™ system. Eligible consenting inpatients will be randomly allocated to either have the ForeSite PT™ system and the LCD monitor turned on (intervention group), or have the ForeSite PT™ system and the LCD monitor turned off (control group) in a ratio of 1:1. Both groups will be included in the study up to a maximum of 7 days. Interface pressure and clinical outcomes will be measured at baseline (within 24 hours of admission) and on the last day of enrollment in the trial (within 24 hours prior to discharge, or on the seventh day (whichever comes first)). The perceptions of patients allocated to the intervention group, and the perceptions of healthcare providers each time a patient in their care is allocated to the intervention group, will be measured on the last day of enrollment in the trial or on the seventh day (whichever comes first).

Participants

Recruitment

Identification of eligible inpatients will be facilitated by the charge nurse, the patient care manager, and nurses on their respective units, and by daily review of new admissions. After an eligible patient has been identified, the Research Nurse or the Research Assistant will ask him or her (or a substitute decision-maker, if not capable of consenting), if he or she would be interested in participating in a study to evaluate a pressure sensing technology that provides real-time feedback to healthcare providers. If the patient agrees to participate, he or she will be asked to provide informed consent. The Research Nurse will set up the ForeSite PT™ system, including placing the sensor overlay under the linen and mounting the LCD monitor to the headwall. The Research Nurse will ensure that the overlay and monitor are functioning properly on a daily basis, and will instruct housekeeping on sterilization practices of these device components.

Eligibility

The selection of participants will be based on the following inclusion and exclusion criteria:

Inclusion criteria:

- Adult, man or woman, with a minimum age limit of 18 years old;
- Expected to have a length of stay on the unit of at least 4 days;
- Require assistance with bed mobility or completely dependent for bed mobility (identified as those who had difficulty shifting themselves independently and who needed assistance with turning within their bed and/or getting in and out of bed);
- Cognitively competent to provide consent, or is cognitively impaired and have a family member provide consent on their behalf; and
- Not near the end of life within three days of enrollment in the study.

Exclusion criteria:

- Have a planned admission to another unit (including those identified as a setting for data collection in the study) within three days of enrollment in the study;
- Sleep in a chair at night; and
- Whose clinical care would be negatively impacted if turned or repositioned.

Setting

This trial will take place at five inpatient care settings at the Foothills Medical Centre, a tertiary care institution, in Calgary, Alberta, Canada. The three inpatient care settings include: 1) Units 36 and 37 in the Special Services Building, the internal medicine services that cares for vulnerable medical inpatients with immobility arising from complex multi-system medical illness; 2) the intensive care unit (ICU) that

cares for sedated and immobilized patients requiring life support for prolonged periods of time; and 3) Unit 112 and Unit 58, the neurological rehabilitation unit that cares for patients with prolonged immobility and vulnerability resulting from spinal cord and/or significant head injuries.

Intervention

All eligible consenting inpatients will receive usual care by their healthcare providers, and undergo interface pressure monitoring by the ForeSite PT™ system.

Treatment Group

Inpatients allocated to the treatment group will have the ForeSite PT™ system's LCD monitor turned on (i.e., real-time images of interface pressure will be displayed on the monitor) during their enrollment in the trial.

Control Group

Inpatients allocated to the control group will have the ForeSite PT™ system's LCD monitor turned off and hidden (i.e., real-time images of interface pressure will not be displayed on the monitor). As the ForeSite PT™ system will continue to sample interface pressure with the display turned off, this enables patients enrolled in the control group to undergo silent monitoring.

Outcomes

Primary Outcome

1. A composite of interface pressure measurements that reflect the distribution of interface pressure at predetermined time intervals. These measures include:
    a. proportion of participants that have pressure readings greater than 40 mmHg;
    b. average interface pressure (excluding sensels with 0mmHg reading); and
    c. the absolute number of sensels with pressure readings greater than 40 mmHg.

The measurement of this outcome will be facilitated by the ForeSite PT™ system's sensor overlay that continuously collects pressure readings. The array of the sensor overlay is a capacitive sensor with a 0.625" spatial resolution. There are 52 rows x 118 columns for 6,136 sensing points. The ForeSite PT™ system's software continuously samples interface pressure at a rate of 1 Hz, which can then be extracted for analysis. The data file is in a proprietary binary format, and can be exported to TXT or CSV formats for import into a spreadsheet program for analysis. This will be achieved by the sampling of pressure readings at specified time intervals before and after turning/repositioning when the ForeSite PT™ system's LCD monitor is turned on, and when it is turned off.

Secondary Outcomes

1. A composite of endpoints that reflect compromised skin and soft tissues, with potential for progression to overt pressure ulceration. This will include:
    a. skin discolouration;
    b. localized tenderness without skin breakdown;
    c. Stage I and II (partial thickness) pressure ulcer formation;
    d. Stage III and IV (full thickness) pressure ulcer formation;
    e. unstageable/Unclassified: Full thickness skin or tissue loss – depth unknown;
    f. suspected Deep Tissue Injury – depth unknown; or
    g. presence of any skin or wound infection (i.e., cellulitis, infected ulcers, or osteomyelitis).

This outcome will be measured by a head-to-toe skin clinical assessment for detection of pressure-related skin changes and for overt ulceration by the Research Nurse. This assessment will be conducted within 24 hours of admission and 24 hours prior to discharge, or on the seventh day (whichever comes first). This information will be noted on a form, which requires the Research Nurse to circle the skin areas where the patient may have skin changes or a pressure ulcer. They will label the circled area with the numbers 1 to 9 that correspond to predetermined categories of pressure-related skin changes and stages of pressure ulcer development.

The presence of a pressure ulcer and its severity, as well as the development of other skin condition changes, will be determined as per the National Pressure Ulcer Advisory Panel's categories/staging definitions (2,3,4,5,6,7) and other skin appearance changes (1,8,9) described below:

**Numbered Labels**	**Descriptions**
**1. Pressure-related blanchable erythema (excluding dermatitis, cellulitis, trauma)**	Intact skin with redness; skin remains blanchable on compression, potentially reversible change
**2. Stage I pressure ulcer (non-blanchable erythema)**	Intact skin with non-blanchable redness of a localized area usually over a bony prominence. Darkly pigmented skin may not have visible blanching; its color may differ from the surrounding area. The area may be painful, firm, soft, warmer or cooler as compared to adjacent tissue. Category I may be difficult to detect in individuals with dark skin tones. May indicate "at risk" persons.
**3. Stage II pressure ulcer (partial thickness skin loss)**	Partial thickness loss of dermis presenting as a shallow open ulcer with a red pink wound bed, without slough. May also present as an intact or open/ruptured serum-filled or sero-sanginous filled blister.Presents as a shiny or dry shallow ulcer without slough or bruising*. This category should not be used to describe skin tears, tape burns, incontinence associated dermatitis, maceration or excoriation.<br>*Bruising indicates deep tissue injury.

**4. Stage III pressure ulcer (full thickness skin loss)**	Full thickness tissue loss. Subcutaneous fat may be visible but bone, tendon or muscle are *not* exposed. Slough may be present but does not obscure the depth of tissue loss. *May* include undermining and tunneling. The depth of a Category/Stage III pressure ulcer varies by anatomical location. The bridge of the nose, ear, occiput and malleolus do not have (adipose) subcutaneous tissue and Category/Stage III ulcers can be shallow. In contrast, areas of significant adiposity can develop extremely deep Category/Stage III pressure ulcers. Bone/tendon is not visible or directly palpable.
**5. Stage IV pressure ulcer (full thickness tissue loss)**	Full thickness tissue loss with exposed bone, tendon or muscle. Slough or eschar may be present. Often includes undermining and tunneling. The depth of a Category/Stage IV pressure ulcer varies by anatomical location. The bridge of the nose, ear, occiput and malleolus do not have (adipose) subcutaneous tissue and these ulcers can be shallow. Category/Stage IV ulcers can extend into muscle and/or supporting structures (e.g., fascia, tendon or joint capsule) making osteomyelitis or osteitis likely to occur. Exposed bone/muscle is visible or directly palpable.
**6. Unstageable/Unclassified: Full thickness skin or tissue loss – depth unknown**	Full thickness tissue loss in which actual depth of the ulcer is completely obscured by slough (yellow, tan, gray, green or brown) and/or eschar (tan, brown or black) in the wound bed. Until enough slough and/or eschar are removed to expose the base of the wound, the true depth cannot be determined; but it will be either a Category/Stage III or IV. Stable (dry, adherent, intact without erythema or fluctuance) eschar on the heels serves as "the body's natural (biological) cover" and should not be removed.
**7. Suspected Deep Tissue Injury – depth unknown**	Purple or maroon localized area of discolored intact skin or blood-filled blister due to damage of underlying soft tissue from pressure and/or ***shear.*** The area may be preceded by tissue that is painful, firm, mushy, boggy, warmer or cooler as compared to adjacent tissue. Deep tissue injury may be difficult to detect in individuals with dark skin tones. Evolution may include a thin blister over a dark wound bed. The wound may further evolve and become covered by thin eschar. Evolution may be rapid exposing additional

	layers of tissue even with optimal treatment.
**8. Infection – cellulitis around pressure ulcer**	This presents as redness, warmth and swelling in the skin around the pressure ulcers.
**9. Infection – pressure ulcer wound base infection**	This presents as drainage (potentially purulent) with strong odour from the base of the pressure ulcer. May have necrotic material as wound base.

2. Perceptions (both positive and negative) of healthcare providers caring for patients allocated to the treatment group to the ForeSite PT™ system. This outcome will be measured by a survey consisting of 17 close-ended and open-ended questions about prior experience with pressure mapping technology, and functionality, ease of use, and interpretation of pressure data on the LCD monitor. These questions were utilized in a prior pilot study assessing healthcare providers' perceptions to the interface pressure information provided on the LCD monitor, and the impact of this information on patient care. The survey will be administered to each healthcare provider once after their first use of the ForeSite PT™ system in the trial by the Research Nurse or the Research Assistant on the patient's last day of enrollment or on the seventh day (whichever comes first), and will take approximately 10 minutes to complete.

3. Perceptions (both positive and negative) of patients (and if appropriate, family members) allocated to the treatment group to the ForeSite PT™ system. This outcome will be measured by a survey consisting of seven close-ended and open-ended questions about prior experience with pressure mapping technology and the ForeSite PT™ system (including sensor overlay and monitor display of their pressure distribution) on their care and comfort. The survey will be administered by the Research Nurse or the Research Assistant, and will take approximately 10 minutes to complete.

In addition, a pressure ulcer risk assessment will be conducted, and demographic information and information on medical history will be collected from patient charts, to establish a patient profile at baseline, within 24 hours of admission, by the Research Nurse or the Research Assistant.

The Braden Scale will be administered to determine the patient's level of risk at pressure ulceration. The Braden Scale was developed to assist healthcare providers with assessing a patient's level of risk for pressure ulcer development and the course of a particular treatment. It is a summated rating scale composed of six subscales: sensory perception, activity, mobility, moisture, friction, and nutrition. Each subscale is scored from 1-3 or 1-4, for a total score that ranges from 6-23. A lower total score indicates a lower level of functioning and therefore a higher level of risk for pressure ulceration. Second, a head-to-toe skin assessment will be conducted for detection of pressure-related skin changes and for overt ulceration. It requires circling skin areas where the patient may have skin changes or a pressure ulcer, and labelling the same area with the numbers 1 to 9, which correspond with stages of skin change and pressure ulceration as per the staging system developed by the National Pressure Ulcer Advisory Panel and other skin appearance changes described above. The research nurse will conduct the Braden Scale

and the head-to-toe skin assessment a second time within 24 hours prior to their discharge date, or on the seventh day (whichever comes first).

The demographic information that will be collected include: sex, date of birth, and race. Information on medical history that will be collected include: Charlston comorbidity index; CBC, pre-albumin and albumin, creatinine, liver function test results (alkaline phosphatase, AST), C reactive protein (CRO), glycosylated hemoglobin, and body mass index (BMI). Patient history data that will be collected include reason(s) for admission/diagnoses, level of bed mobility, bladder management, bowel management, history of cancer and/or treatment, history of cardiovascular disease, history of renal failure, use of immunosuppressive medications, previous pressure ulcers, smoking history, and illicit drug use.

4. Total cost of per admission. This information will be used in determining the cost-effectiveness of the ForeSite PT™ system. This information will be accessed using the participant's personal health number after he or she has been discharged from the hospital.

Sample size

A total of 600 patients will be recruited from Unit 36, Unit 58, and the ICU will be enrolled in the trial, in order to randomize 300 patients to each of the intervention and control arms. Sample size estimates for the primary outcome indicate a need for 294 patients in each of the intervention and control arms. This is based on an estimated control group event rate of 30%, an intervention group rate of 20% (i.e., a 33% relative risk reduction), an alpha level of 0.05, and 80% power.

Randomization

The random allocation of patients to intervention vs. control groups will be conducted following enrollment in the trial (i.e., admission to study settings). Allocation will be completed in a concealed manner by a research methodologist in the Calgary Institute for Population and Public Health at the University of Calgary, using a random number generator that will produce a randomization list organized in blocks of 6.

The list will be used to prepare sealed, opaque, allocation envelopes that will be stored in a secure documents cabinet in the clinical setting. The Research Nurse and Research Assistant will then access these envelopes in the secure storage area to determine patient allocation at the time of randomization.

Blinding

The researchers, who will be involved in data analysis, including the Principle Investigator, Co-Investigators, and the Research Coordinator, will be blinded to treatment allocation. The Research Nurse and the Research Assistant will both be involved in participant recruitment, the randomization process, and data collection, and will not be blinded to treatment allocations. It will be impossible to blind

participants and healthcare providers as they will be able to see if the LCD monitor that displays information about the patient's surface pressure on the bed is mounted to headwall.

Analysis and Statistical Methods

Subjects will be stratified by their pressure ulcer risk, as determined by their Braden Score.

Data analysis will involve use of Chi-square and/or Fisher exact tests for between-group (i.e., intervention and control groups) comparisons of the primary outcome and the secondary outcomes, and the Wilcoxon rank sum test for between-group comparison of strategic shifting. Chi square and t-tests will be used to compare baseline characteristics between groups, and logistic regression will be used for adjusted analyses if there are notable between-group differences for important baseline characteristics. Correlation between pressure distribution (the study's primary endpoint) with the hard clinical outcome (pressure ulceration and pressure-related skin appearance changes) will also be explored.

Univariate statistics will be used to summarize changes in pressure distribution as per the interface pressure feedback provided by the ForeSite PT™ system.

Univariate statistics will be used to summarize close-ended survey data, while open-ended survey data will be thematically analyzed.

Economic Evaluation

An economic evaluation will be conducted in addition to this randomized controlled trial to determine the cost per pressure ulcer event avoided from a health system payer perspective. This will involve the compilation of information on device costs, operational and maintenance costs, human resource requirements, and costs of care associated with developing a pressure ulcer. The resulting cost per pressure ulcer event avoided will indicate the value-for-money for health systems associated with the ForeSite PT™ system.
