# Supplementary material for: Effect of a Continuous Bedside Pressure Mapping System for Reducing Interface Pressures: A Randomized Clinical Trial
Source: JAMA Netw Open. 2023 Jun 2;6(6):e2316480. doi: 10.1001/jamanetworkopen.2023.16480 (PMC10238950; doi:10.1001/jamanetworkopen.2023.16480)
Supplement: Supplement 2. — eTable 1. Other Baseline Characteristics of Control vs. Intervention Groups eTable 2. Baseline Characteristics of Patients Included vs. Patients Included in Interface Pressure Analysis eTable 3. Non-Pre-Specified Subgroup Analysis of Primary and Secondary Outcomes Stratified by ICU vs. Non-ICU Location of Care [file jamanetwopen-e2316480-s002.pdf]

Supplemental Online Content

Ho C, Ocampo W, Southern DA, et al. Effect of a continuous bedside pressure mapping system for reducing interface pressures. *JAMA Netw Open.* 2023;6(6):e2316480. doi:10.1001/jamanetworkopen.2023.16480

eTable 1. Other Baseline Characteristics of Control vs. Intervention Groups

eTable 2. Baseline Characteristics of Patients Included vs. Patients Included in Interface Pressure Analysis

eTable 3. Non-Pre-Specified Subgroup Analysis of Primary and Secondary Outcomes Stratified by ICU vs. Non-ICU Location of Care

This supplemental material has been provided by the authors to give readers additional information about their work.

© 2023 Ho C et al. *JAMA Network Open.*

eTable 1. Other baseline characteristics of control vs. intervention groups

Baseline Characteristics	Control Monitor OFF N=260	Intervention Monitor ON N=247
**Age**		
**<40 years**	36 (13.9)	29 (11.7)
**40-64 years**	87 (33.5)	86 (34.8)
**65-74 years**	70 (26.9)	57 (23.1)
**75-84 years**	45 (17.3)	44 (17.8)
**85+ years**	22 (8.5)	31 (12.6)
**Fitzpatrick Skin Type**		
**1**	21 (8.1)	19 (7.7)
**2**	169 (64.8)	182 (73.7)
**3**	47 (18.0)	31 (12.6)
**4**	18 (6.9)	9 (3.6)
**5**	3 (1.2)	2 (0.8)
**6**	0	1 (0.4)
**Missing/not entered**	3 (1.2)	3 (1.2)
**History of**		
**Cancer Diagnosis**	51 (19.5)	52 (21.1)
**Cancer Treatment**	48 (18.4)	51 (20.7)
**Cardiac Diagnosis**	117 (44.8)	111 (44.9)
**Renal Failure**	38 (14.6)	34 (13.8)
**Immunosuppressive meds**	32 (12.3)	30 (12.2)
**Diabetes**	82 (31.4)	66 (26.7)
**Pressure Injury**	21 (8.1)	23 (9.3)
**Former Smoker**	114 (43.7)	122 (49.4)
**Former Illicit Drug User**	17 (6.5)	17 (6.9)
**Status at admission**		
**Cardiac Disease**	115 (44.1)	109 (44.1)
**Renal Failure**	62 (23.8)	59 (23.9)
**Pressure Injury**	34 (13.0)	25 (10.1)
**Current Smoker**	43 (16.5)	44 (17.8)
**Current Illicit Drug User**	12 (4.6)	13 (5.3)
**Urinary Incontinence**	246 (94.3)	230 (93.1)
**Bowel Incontinence**	233 (89.3)	215 (87.0)
**Reason for Admission**		
**Respiratory**	52 (19.9)	67 (27.1)
**Cardiology**	23 (8.8)	21 (8.5)
**Neurologic**	100 (38.3)	78 (31.6)
**GI**	10 (3.8)	23 (9.3)
**Vascular**	27 (10.3)	21 (8.5)
**Renal**	26 (10.0)	20 (8.1)
**Hepatic**	7 (2.7)	13 (5.3)

© 2023 Ho C et al. *JAMA Network Open.*

**Trauma**	36 (13.8)	33 (13.4)
**Diabetes**	8 (3.1)	6 (2.4)
**Cancer**	8 (3.1)	12 (4.9)
**Sepsis**	33 (12.6)	38 (15.4)
**Surgical**	30 (11.5)	32 (13.0)
**Other**	59 (22.6)	43 (17.4)
**BRADEN SCORE**		
**Sensory Perception**		
**Completely Limited**	35 (13.5)	28 (11.3)
**Very Limited**	73 (28.1)	62 (25.1)
**Slightly Limited**	112 (43.1)	110 (44.5)
**No Impairment**	40 (15.4)	47 (19.0)
**Moisture**		
**Very Moist**	22 (8.5)	13 (5.3)
**Constantly Moist**	3 (1.2)	6 (2.4)
**Occasionally Moist**	158 (61.0)	153 (61.9)
**Rarely Moist**	76 (29.3)	75 (30.4)
**Activity**		
**Bedfast**	162 (62.3)	177 (71.7)
**Chairfast**	93 (35.8)	67 (27.1)
**Walks Occasionally**	5 (1.9)	3 (1.2)
**Mobility**		
**Completely Immobile**	75 (28.9)	62 (25.1)
**Very Limited**	137 (52.7)	137 (55.5)
**Slightly Limited**	48 (18.5)	47 (19.0)
**No Limitation**	0	1 (0.4)
**Nutrition**		
**Very Poor**	17 (6.5)	12 (4.9)
**Probably Inadequate**	60 (23.1)	80 (32.3)
**Adequate**	181 (69.6)	155 (62.8)
**Excellent**	2 (0.8)	0
**Friction and Shear**		
**Problem**	72 (27.7)	57 (23.1)
**Potential Problem**	183 (70.4)	189 (76.5)
**No Apparent Problem**	5 (1.9)	1 (0.4)

© 2023 Ho C et al. *JAMA Network Open.*

eTable 2. Baseline characteristics of patients included vs. patients included in interface pressure analysis

Baseline Characteristics	Excluded N=171	Included N=507
**Average Age (Std Dev)**	61.5 (19.6)	63.0 (17.9)
**Male**	96 (56.1)	299 (59.0)
**Bed Mobility**		
**Dependent**	107 (62.6)	323 (63.7)
**Non-dependent**	62 (36.3)	182 (35.9)
**Missing/not entered**	2 (1.2)	2 (0.4)
**Mean BMI (std dev)**	34.9 (35.3)	30.9 (14.5)
**Average Weight (std dev)**	87.0 (32.8)	87.0 (26.8)
**Charlson Comorbidities**		
**AIDS/HIV**	0	2 (0.4)
**CEVD**	73 (43.2)	222 (44.1)
**COPD**	26 (15.4)	108 (21.4)
**CHF**	30 (17.7)	120 (23.8)
**MI**	24 (14.1)	71 (14.1)
**Dementia**	10 (5.9)	45 (8.9)
**PVD**	23 (13.5)	75 (14.8)
**Paraplegia**	58 (34.3)	153 (30.2)
**Leukemia**	4 (2.4)	7 (1.4)
**Lymphoma**	3 (1.8)	7 (1.4)
**PUD**	29 (17.3)	82 (16.3)
**Renal Disease**	30 (17.8)	102 (20.2)
**Liver Disease**	13 (7.7)	39 (7.8)
**Diabetes**	38 (22.4)	153 (30.3)
**Solid Tumor**	23 (13.5)	96 (19.1)
**Connective tissue Disease**	27 (15.9)	82 (16.4)
**Lab Values**		
**Average Hgb (std dev)**	105.8 (22.1)	102.2 (21.9)
**Average HCT (std dev)**	0.3 (0.1)	0.6 (6.4)
**Average RBC (std dev)**	3.5 (0.9)	3.6 (3.8)
**Average Platelet (std dev)**	237.5 (123.8)	243.8 (138.1)
**Average Creatinine (std dev)**	111.1 (117.0)	114.7 (116.7)
**Average WBC (std dev)**	13.6 (18.6)	12.0 (13.2)
**BRADEN SCORE**	13.48 (2.07)	13.40 (2.07)
**Pressure Injury Status**		
**No PI at baseline**	109 (63.7)	302 (59.6)
**PI positive at baseline**	62 (36.3)	205 (40.4)
**Number of areas at baseline**		
**1**	26 (41.9)	113 (55.1)
**2**	23 (37.1)	45 (22.0)
**3**	8 (12.9)	30 (14.6)
**4+**	5 (8.1)	17 (8.3)
**Intervention**		
**Monitor ON**	92 (27.2)	246 (72.8)
**Monitor OFF**	79 (23.2)	261 (76.8)

© 2023 Ho C et al. *JAMA Network Open.*

eTable 3. Non Pre-specified Subgroup Analysis of Primary and Secondary Outcomes stratified by ICU vs. non-ICU Location of Care.

Primary Outcomes	ICU			Non-ICU		
	Control Monitor OFF N=96	Intervention Monitor ON N=95	p-value	Control Monitor OFF N=164	Intervention Monitor ON N=152	p-value
Peak pressure 72 hours	256.0	256.0	-	256.0	256.0	-
Absolute number of sensels with pressure readings > 40 mmHg, No.	4,389	4,095	0.15 [a]	6,644	5,219	0.16 [a]
Pressure in mmHg, Mean (SD)	6.93 (1.79)	6.78 (1.45)	0.54 [b]	6.73 (1.53)	6.51 (1.54)	0.20 [b]
Proportion of participants that have pressure readings > 40 mmHg, N (%)	96 (100.0)	95.0 (100.0)	-	163 (99.4)	151 (99.3)	0.96 [b]
Count of Sensels with pressure>40 mmHg, Median (IQR)	33.67 (13.5)	32.08 (17.9)	0.99 [c]	26.4 (23.5)	21.1 (12.5)	0.13 [c]
Pressure in mmHg, Median (IQR)	6.58 (0.90)	6.43 (0.84)	0.64 [c]	6.54 (0.89)	6.15 (0.85)	0.08 [c]
**Secondary Outcomes**	**ICU**			**Non-ICU**		
	**Control Monitor OFF N=96**	**Intervention Monitor ON N=95**	**p-value**	**Control Monitor OFF N=164**	**Intervention Monitor ON N=152**	**p-value**
No Pressure Injury at baseline, No.	*61*	*73*		*88*	*79*	
Pressure Injury at 72 hrs, No. (%)	9 (14.8)	15 (20.6)	0.38 [a]	14 (15.9)	16 (20.3)	0.47 [a]
Pressure Injury at baseline & at 72 hrs, No.	*35*	*22*		*30*	*23*	
New location at 72 hrs, No. (%)	7 (20.0)	5 (22.7)	0.81 [a]	22 (29.0)	5 (6.9)	0.0005 [a]

Note: Only non-zero values included in calculations; ; [a] Chi-Square; [b]F-test;[c]Kruskal-Wallis Test

© 2023 Ho C et al. *JAMA Network Open.*
