# Supplementary material for: Effect of a Continuous Bedside Pressure Mapping System for Reducing Interface Pressures: A Randomized Clinical Trial
Source: JAMA Netw Open. 2023 Jun 2;6(6):e2316480. doi: 10.1001/jamanetworkopen.2023.16480 (PMC10238950; doi:10.1001/jamanetworkopen.2023.16480)
Supplement: Supplement 3. — Data Sharing Statement [file jamanetwopen-e2316480-s003.pdf]

Data Sharing Statement

Ho. Effect of a Continuous Bedside Pressure Mapping System for Reducing Interface Pressures. *JAMA Netw Open*. Published June 02, 2023.
doi:10.1001/jamanetworkopen.2023.16480

Data
Data available: Yes
Data types: Deidentified participant data, Data dictionary
How to access data: Data can be made available with a data transfer agreement and can be accessed using a secure University of Calgary external shared folder. You can email w21c@ucalgary.ca or chester.ho@albertahealthservices.ca
When available: With publication

Supporting Documents
Document types: None

Additional Information
Who can access the data: Researchers whose proposed use of the data has been approved
Types of analyses: For a specified purpose
Mechanisms of data availability: With a signed data access agreement
